# USC ALZHEIMER’S DISEASE AND ALZHEIMER’S DISEASE-RELATED DEMENTIAS (RCMAR) CENTER: LESSONS LEARNED

**DOI:** 10.1093/geroni/igae098.0681

**Published:** 2024-12-31

**Authors:** Jennifer Ailshire

**Affiliations:** University of Southern California, Los Angeles, California, United States

## Abstract

The National Institute on Aging has awarded another five-year, $3.6M grant to fund the University of Southern California Alzheimer’s Disease and Alzheimer’s Disease-Related Dementias Resource Center for Minority Aging Research. The Center is led by the multidisciplinary team of Julie Zissimopoulos, Jennifer Ailshire, and Emma Aguila. The USC AD/ADRD RCMAR supports junior scientists exploring health and economic disparities associated with dementia. The RCMAR – housed within the USC Schaeffer Center for Health Policy & Economics – focuses on pathways by which social, behavioral, and economic factors – as well as policies and health systems – affect disparities in risk of dementia and disparities in the health, health care and economic outcomes of persons living with dementia. The center also helps launch the careers of underrepresented junior investigators, providing funding, mentorship, and tools to support their pilot research projects. The Center was established in 2012 and has supported 34 scholars from USC and 14 other institutions. Alumni of the program have received more than $11.2 million in research funding and have published more than 268 peer-reviewed articles. Many scientists have received academic promotions and met career goals. The Center has an extensive network of supporters and collaborators including Howard University, Spelman College, Cal State Fullerton and other NIA-funded centers at USC including: Roybal Center for Behavioral Interventions of Aging, Alzheimer Disease Research Center, Center for Economic and Sociodemographic Study of AD/ADRD, and USC/UCLA Center on Biodemography and Population Health. The Center also supports the annual Science of AD/ADRD for Social Scientists.

